# Light flash and odor during proton beam therapy for pediatric patients: a prospective observational study

**DOI:** 10.3389/fonc.2022.863260

**Published:** 2022-08-01

**Authors:** Masashi Mizumoto, Yoshiko Oshiro, Toshio Miyamoto, Taisuke Sumiya, Keiichiro Baba, Motohiro Murakami, Shosei Shimizu, Takashi Iizumi, Takashi Saito, Hirokazu Makishima, Haruko Numajiri, Kei Nakai, Toshiyuki Okumura, Kazushi Maruo, Takeji Sakae, Hideyuki Sakurai

**Affiliations:** Department of Radiation Oncology, University of Tsukuba, Tsukuba, Japan

**Keywords:** light flash, odor, proton beam therapy, pediatric, prospective

## Abstract

Light flash and odor during radiation therapy are well-known phenomena, but the details are poorly understood, particularly in pediatric patients. Therefore, we conducted a prospective observational study of these events in pediatric patients (age ≤20 years old) who received radiotherapy at our center from January 2019 to November 2021. Light flash and odor were evaluated using a patient-reported checklist including the presence, strength, and duration of the phenomenon, and color of light or type of odor. 53 patients who received proton therapy (n=47) and photon radiotherapy (n=6) were enrolled in this study. The median age of the patients was 10, ranged from 5 to 20. The patients who was able to see the light flash was 4, and all of them received retina irradiation. This was equivalent to 57% of the patients who received radiotherapy to retina (n=7). The light was bright and colored mainly blue and purple, which seemed to be consistent with Cherenkov light. Odor was sensed by 9 (17%) patients, and seven patients of the 9 received nasal cavity irradiation. This was equivalent to 41% of the patients who received nasal cavity irradiation (n=17). Other 2 patients received proton therapy to brain tumor. The odors were mainly described as plastic, burnt and disinfectant, which may be caused by ozone generated during irradiation. These data suggest that pediatric patients with retinal and nasal cavity irradiation frequently sense light flashes or odor. So adequate care is necessary so that these patients are not worried about this phenomenon.

## Introduction

Light flash or odor during radiation therapy is a well-known phenomenon among radiation oncologists, but has not been evaluated in detail because it does not cause harm to the patient (1–7). Cherenkov light is thought to be the main cause of the light flash during irradiation, but no clear conclusion has been reached (8–10). A trace amount of ozone generated by radiation therapy is likely to be the main cause of odor during radiation therapy, but it is difficult to detect this small amount of ozone and similarly there is no clear conclusion on the source of the odor (1, 4).

Light flash and odor during irradiation may mainly occur in radiotherapy for brain or head and neck tumors (1–4). In our previous survey of light flash and odor during irradiation using a common checklist, we found that light flash was experienced by 55% of adults if the retina was included in the irradiation range, but only by 7% if the retina was not in this range (4). Odor during radiotherapy occurred in 27% of cases in which the nasal cavity was included in the irradiation range, but only in 5% of cases in which irradiation did not include the nasal cavity (unpublished data).

The survey results also showed that younger people were more likely to be aware of light flash and odor during irradiation, but there were only a few pediatric patients (under 20 years old) included in the study and the details could not be evaluated. Therefore, we conducted a prospective observational study on light flash and odor during irradiation in patients under 20 years old.

## Patients and methods

The study was performed from January 2019 to November 2021. This study involving human participants were reviewed and approved by Tsukuba Clinical Research & Development Organization. The participants provided written informed consent to participate in this study. All pediatric patients (≤20 years old) who received proton or photon therapy in the study period were included, except for those who could not communicate and those with abnormal visual or olfactory sensations. Evaluations were made for the presence or absence of light flash or odor during irradiation. Light flash and odor were evaluated using a survey checklist that was completed each week (4).

**Figure 1:**
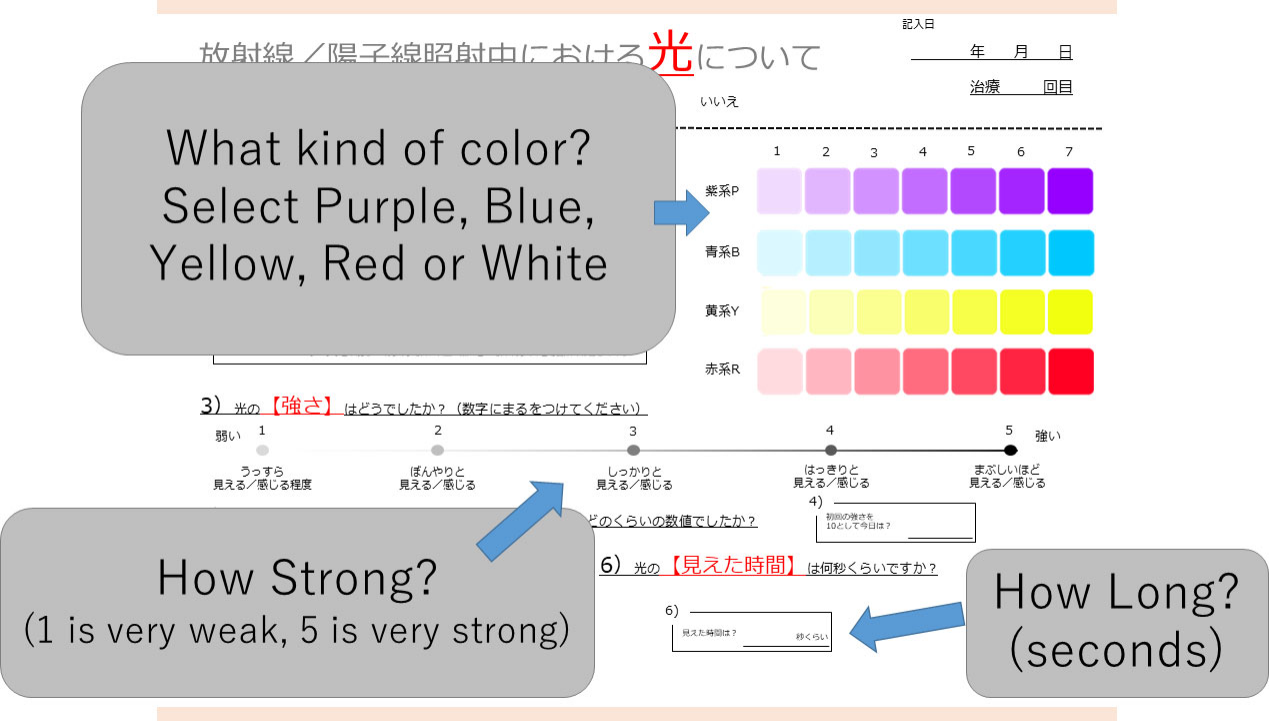
Checklist for evaluation of light flash during irradiation. Patients first indicate if they experienced a light flash. If this answer is “yes”, the color (purple, blue, yellow, red, white), intensity (1 to 5), and time (seconds) are entered by the patient.

The checklist for light flashes (**Figure 1**) requested answers to the following questions: 1 Sense of light flashes (Yes/No); 2 What color? (Purple/Blue/Yellow/Red); 3 How strong? (1-5, 1 very weak, 3 moderate, 5 very strong); 4 How long did you sense light flashes during irradiation?

**Figure 2:**
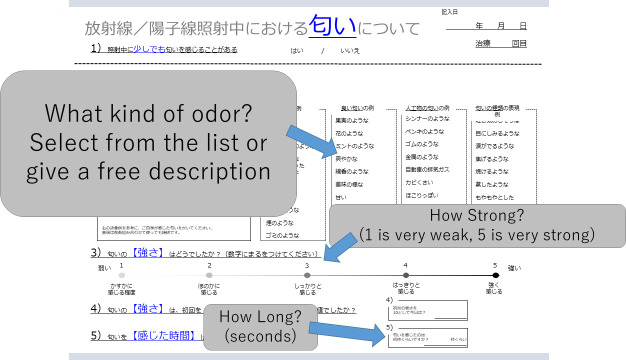
Checklist for evaluation of odor during irradiation. Patients first indicate if they detected an odor. If this answer is “yes”, the kind of odor (selected from a list or provided as a free description), intensity (1 to 5), and time (seconds) are entered by the patient.

The checklist for odor during irradiation (**Figure 2**) included the following items: 1 Sense of odor (Yes/No); 2. What kind of odor? (Unpleasant: Fishy, Livestock, Toilet, Putrid, Tobacco, Sweaty, Sulfur, Smoke, Garbage/Pleasant: Fruit, Flower, Mint, Fresh, Incense, Condiment, Sweet, Sour, Not irritating/Artificial: Paint thinner, Paint, Rubber, Metal, Exhaust gas, Mold, Dust, Propane gas, Alcohol, Bleach/Others: Nauseous, Eye burn, Burn, Steamy, Misty, Clear, Clinging, Messy/Free entry); 3. How strong was the odor? (1-5: 1 very weak, 3 moderate, 5 very strong); 4. How long did the odor last?

The dose-rate of photon therapy was 400-600 MU/min and that of proton therapy was 1300 MU/min. Passive scattering proton therapy with pulsed beam is used in our institution, and our synchrotron basically irradiates with a 0.3-second wide pulse once every 2 seconds, with the dose rate of 1-2 Gy per minutes.

## Statistical analysis

Multivariate logistic regression models were applied for the presence or absence of light flash and odor. Gender, age (>20, ≤20), retina or nasal cavity dose (maximum irradiation dose for either eye or maximum irradiation dose for nasal cavity), and radiotherapy method (proton, other) were included as explanatory variables in the models. In this study, olfactory apparatuses outside the nasal cavity, such as olfactory bulb were not included in nasal cavity. Statistical significance was defined as p<0.05.

## Results

If patients received radiation therapy to multiple sites on the same day, this was counted as one course. Whereas, some patients received multiple courses of radiation therapy during the study period, and thus, a total of 53 courses for 49 patients were examined. Of the 53 courses, 47 were proton therapy and 6 were photon radiotherapy. The patient characteristics are shown in **Table 1**. The median age of the patients was 10, ranged from 5 to 20. Among the 47 courses of proton beam therapy and 6 of photon radiotherapy, there were 4 (8%) reports of light flashes in 4 patients and 9 (17%) of odor in 9 patients.

**Table 1:** Patient characteristics.

Characteristics	Number
Age (years)	5-20 (range) 10 (median)
Gender	
Male	28
Female	21
Irradiated Site	
Brain	27
Head and Neck	2
Chest	7
Abdomen	6
Pelvis	6
Limbs	1
Radiotherapy Technique	
Proton	43
Proton and Photon	4
Photon	2

Of the 4 patients, who experienced light flashes, each 2 patients received proton and photon radiotherapy, and all these patients received retina irradiation (**Table 2**). Retina was included 5 and 2 courses of photon and proton therapy. The colors of these light flashes were blue (n=2), purple (n=1) and yellow (n=1); the intensities (1: very weak, 5: very strong) were 2 (n=2) and 3 (n=2), and the period of the light flashes ranged from 1 to 5 seconds.

**Table 2:** Details of abnormal sensations in each patient.

Patient	Age	Gender	Radiotherapy	Retina	Nasal Cavity	Light Flash	Odor
A	19	F	Photon	100	100	None	Plastic/4/3
B	7	F	Proton	0	0	None	Plastic/1/2
C	16	M	Photon	100	100	Blue/3/1	None
D	18	M	Photon	100	100	Yellow/3/4	Disinfectant/4/4
E	19	F	Proton	60	100	Blue/2/5	Disinfectant/3/10
F	7	F	Proton	0	0	None	Unexplained/3/60
G	11	M	Proton	0	60	None	Disinfectant/3/10
H	12	F	Proton	0	100	None	Sulfur/2/3
I	9	M	Photon	100	100	None	Unexplained/3/3
J	19	M	Proton	100	20	Purple/2/3	None
K	20	M	Proton	0	100	None	Burnt/2/30

Retina: maximum dose to either retina (%), Nasal Cavity: maximum dose to nasal cavity (%), Light Flash: color/intensity/time, Odor: type/intensity/time (s)

Nine patients sensed odor. The patient characteristics are shown in **Table 2**. Of the 53 courses, nasal cavity was included in 17 courses for the 17 patients. Of the 9 patients who sensed odor, 7 patients of the 17 (41%) received nasal cavity irradiation. Other 2 patients received proton beam therapy to brain tumor. The types of odor were disinfectant (n=3), plastic odor (n=2), sulfur odor (n=1), burnt odor (n=1) and unexplainable odor (n=2); the intensities (1: very weak, 5: very strong) ranged from 1 to 4 (median 3), and the time over which the odor persisted was 2-60 seconds (median 4 seconds).

Four patients in the study (**Table 3**) received photon radiotherapy over a wide area, followed by a local proton beam therapy boost. In 3 of the 4 cases, proton beam therapy boost was performed after whole-brain photon irradiation, and in 2 of these 3 cases, light flash or odor was sensed during photon irradiation, but not during the boost therapy, which did not include the retina or nasal cavity in the irradiation range. In the other case, in which the entire lung was irradiated with photon radiotherapy and then boosted with proton therapy for obvious lesions, there was no awareness of light flash or odor during photon radiotherapy or proton beam therapy.

**Table 3:** Details of abnormal sensations in patients who received multiple courses of radiotherapy.

Patient	Age	Gender	Radiotherapy	Retina	Nasal Cavity	Light Flash	Odor
1	16	M	Photon	100	100	Blue/3/1	None
			Proton	0	0	None	None
II	7	M	Photon	100	100	None	None
			Proton	0	0	None	None
III	6	F	left	0	0	None	None
			Proton	0	0	None	None
IV	9	M	Photon	100	100	None	Unexplained/3/3
			Proton	0	0	None	None

Retina: maximum dose to either retina (%), Nasal Cavity: maximum dose to nasal cavity (%), Light Flash: color/intensity/time, Odor: type/intensity/time (s).

The results from the comparison of the current results and our previous results are shown in **Table 4**. Among the patients who received retina irradiation, light flash was observed in 57%, 79%, and 51% of the pediatric patients received photon or proton therapy, adult patients with proton therapy, and adult patients with photon therapy, respectively, whereas, 0% (pediatric), 0% (adult, photon) and 10% (adult, proton) among patients without retina irradiation.

**Table 4:** Comparison of results for pediatric and adult patients.

Patients	Light Flash (n)	Light Flash (%)
Children: Proton, retina irradiated	2/2	100
Adult: Proton, retina irradiated	15/19	79
Adult: Photon, retina irradiated	40/79	51
Children: Proton, retina not irradiated	0/45	0
Adult: Proton, retina not irradiated	0/186	0
Adult: Photon, retina not irradiated	33/337	10
	Odor (n)	Odor (%)
Children: Proton, nasal cavity irradiated	4/12	33
Adult: Proton, nasal cavity irradiated	7/20	35
Adult: Photon, nasal cavity irradiated	20/85	24
Children: Proton, nasal cavity not irradiated	2/35	6
Adult: Proton, nasal cavity not irradiated	0/185	0
Adult: Photon, nasal cavity not irradiated	23/330	7

Among the patients who received nasal cavity irradiation, the odor was sensed in the 33% (pediatric), 35% (adult, photon), and 24% (adult, proton), respectively, and odor was sensed only 6% (pediatric), 0% (adult, photon) and 7% (adult, proton) in the patients without nasal cavity irradiation, respectively.

According to multiple logistic regression analysis, retina dose (OR=1.034, p<0.001) and photon radiotherapy (OR=0.479, p=0.0258) were identified as significant factors of light flash (**Table 5**), and dose to the nasal cavity (OR=1.022, p<0.001), photon radiotherapy (OR=0.444, p=0.0336) and younger age (OR=0.370, p<0.0448) were identified as significant factors of odor (**Table 6**).

**Table 5:** Multivariate logistic regression analysis of presence of a sense of Light Flash.

Variable	Odds ratio	95% CI	p value
Gender (female/male)	1.416	0.822-2.438	0.2099
Age (≤20/>20)	2.477	0.670-9.164	0.1741
Retina dose	1.034	1.028-1.041	<0.001
RT methods (proton/photon)	0.479	0.251-0.915	0.0258

**Table 6:** Multivariate logistic regression analysis of presence of a sense of odor.

Variable	Odds ratio	95% CI	p value
Gender (female/male)	1.546	0.862-2.774	0.1436
Age (≤20/>20)	0.370	0.140-0.977	0.0448
Nasal cavity dose	1.022	1.016-1.028	<0.001
RT methods (proton/photon)	0.444	0.210-0.939	0.0336

## Discussion

In this study, pediatric patients with a median age of 10 years experienced light flashes and odor during irradiation at similar rates to those in adults. Light flashes and odor are patient-reported subjective symptoms, which prevented inclusion of patients under 4 years old in this study. However, for younger patients who can explain light flashes and odor, the checklist used in the study was feasible. Our analysis in adults and previous reports indicate that light flash and odor are associated with irradiation of the head and neck regions, and particularly irradiation of the retina for light flashes and of the nasal cavity for odor. Pediatric patients showed the same tendencies. The small number of pediatric cases prevented multivariate analysis, but as in the analysis in adults, the light flashes were mostly blue or purple and the odor was mainly described as plastic, burnt and disinfectant.

JASTRO/JSHO guidelines and ASTRO model policy recommend proton therapy for pediatric tumors to reduce secondary cancers and late adverse events (11, 12). Since our hospital uses proton therapy for pediatric tumors (13–18), most of the current analysis involved proton therapy, with only a few cases treated with photon radiotherapy. Three cases received whole-brain irradiation with photon radiotherapy and then a local boost with proton beam therapy (**Table 3**, patients I, II and IV). In the whole-brain irradiation, light flash or odor occurred in 2 of these cases, but then disappeared after the switch to proton beam therapy, in which the retina and nasal cavity were out of the irradiation range. These findings indicate that an evaluation using our checklist accurately reflects changes that are due to a change in the irradiation method.

Cherenkov light is thought to be one of the causes of light flash during irradiation, and there are many reports of detection of Cherenkov light during radiation therapy (19–22). Cherenkov light is a pale light, and the light detected in our analyses in pediatric and adult patients was mainly blue and purple, which is consistent with Cherenkov light as the cause of the light flash (4). There is less information about odor, but this may be caused by a small amount of ozone generated by irradiation. Ozone is often described as having a burnt or chemical odor, and these odors were common in adult and pediatric patients, which is consistent with ozone being the cause of the odor (23, 24). Nara et al. recently measured the ozone concentrations in the treatment room (25). However, they also showed that ozone levels in the treatment room were undetectable before the start of daily treatment but reached detectable 1 hour after the start of treatment. Ozone is an unstable substance and disappears in a short time, so the odor sensing rate may increase when the nasal cavity was included in the irradiated field. Our recent research showed the patients who received total body irradiation (TBI) felt odor (14 of 32 patients) and 2 of 14 patients answered “ozone” as the type of smell without knowledge of ozone as a possible cause (26). These reports indicate that ozone may be cause of the odor, however various types of odors have been reported in these reports as well (26, 27), and it is not possible to identify the cause of the odors as ozone alone.

It is difficult to determine the exact causes of light flash and odor during irradiation, but the results of this study and our previous studies suggested a relationship with irradiation around the retinal or nasal cavity in all patients with photon radiotherapy or proton beam therapy. Collectively, the data suggest that ≥50% of patients with retina irradiation will sense light flashes, and that about 30% with irradiation of the nasal cavity will experience odor. Some children may feel anxious or afraid of these phenomena. It will be necessary to explain these phenomena carefully when there are questions about them so that t so that they will not feel uneasy.

## Data availability statement

The original contributions presented in the study are included in the article/supplementary material. Further inquiries can be directed to the corresponding author.

## Ethics statement

This study involving human participants were reviewed and approved by Tsukuba Clinical Research & Development Organization. The participants provided written informed consent to participate in this study.

## Author contributions

Conception and design of the study MaM, YO. Analysis and interpretation of data KM. Collection and assembly of data MaM, YO, TM, TSu, KB, MoM, SS, TI, TSa, HM, HN, KN, TO. Drafting of the article MaM, YO, TS, KM. Critical revision of the article for important intellectual content YO. Final approval of the article HS. All authors contributed to the article and approved the submitted version.

## Funding

This work was supported by the University of Tsukuba.

## Conflict of interest

The authors declare that the research was conducted in the absence of any commercial or financial relationships that could be construed as a potential conflict of interest.

## Publisher’s note

All claims expressed in this article are solely those of the authors and do not necessarily represent those of their affiliated organizations, or those of the publisher, the editors and the reviewers. Any product that may be evaluated in this article, or claim that may be made by its manufacturer, is not guaranteed or endorsed by the publisher.
